# Hernie interne supravésicale, cause rare d'occlusion intestinale aiguë

**DOI:** 10.11604/pamj.2015.21.14.6735

**Published:** 2015-05-07

**Authors:** Harissou Adamou, Oumarou Habou, Ousseini Adakal, Ibrahim Amadou Magagi

**Affiliations:** 1 Service de Chirurgie Générale, Hôpital National de Zinder, Zinder, Niger; 2 Service de Chirurgie Pédiatrique, Assistant à la Faculté des Sciences de la Santé de Zinder, Zinder, Niger; 3 Service de Chirurgie Générale, Assistant à la Faculté des Sciences de la Santé de Maradi, Maradi, Niger

**Keywords:** Hernie supravésicale, occlusion intestinale, Niger, Supra vesical hernia, bowel obstruction, Niger

## Abstract

La hernie interne supravésicale est rare et se développe au niveau de la fossette supravésicale. Son diagnostic est très souvent fait à l'occasion d'une laparotomie pour occlusion intestinale. Dans ce travail, nous rapportons le cas d'un patient âgé de 49 ans admis aux urgences chirurgicales de l'HNZ pour syndrome occlusif. La laparotomie réalisée a permis de découvrir une anse grêle incarcérée dans une hernie interne supravésicale. Le geste chirurgical a consisté en une résection du sac associée à une fermeture en points séparés et les suites opératoires ont été simples. Devant toute occlusion intestinale aiguë, il faut avoir à l'esprit que la hernie interne supravésicale peut en être une cause inhabituelle.

## Introduction

Décrite pour la première fois en 1804 par Sir Astley Cooper [1], la hernie interne supravésicale est inhabituelle et peu rapportée dans la littérature [1–9]. Révélée souvent par une complication, la hernie interne supravésicale pose un problème diagnostique pour les cas simples. Elle est très souvent découverte à l'occasion d'un syndrome occlusif. Nous rapportons l'observation de la prise en charge d'un patient porteur d'une hernie supravésicale interne révélée par une occlusion intestinale aiguë.

## Patient et observation

**Figure 1:**
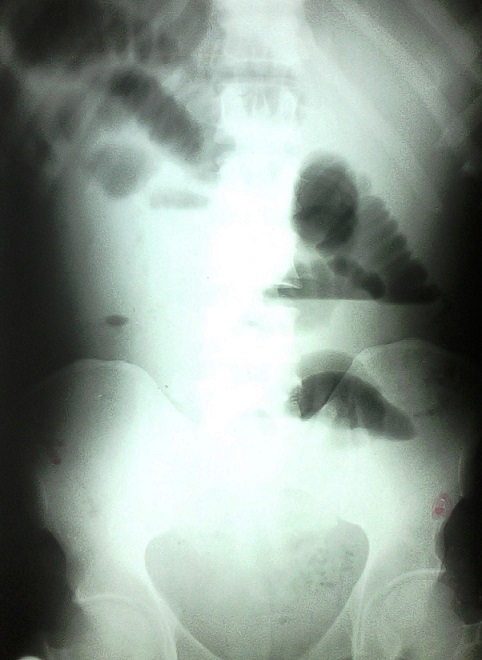
Radiographie de l'abdomen sans préparation montrant des images en faveur d'une occlusion intestinale

**Figure 2:**
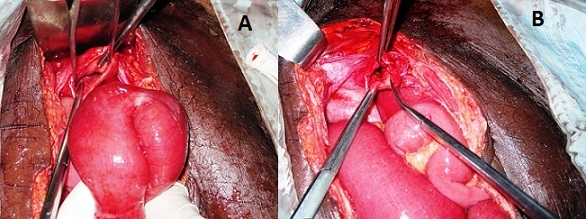
(A) orifice herniaire supravésical, à coté une anse grêle viable après libération; (B) résection partielle du péritoine pelvien constituant le sac herniaire avant la suture

Patient âgé de 49 ans aux antécédents de douleurs abdominales à type de coliques, était admis aux urgences dans un tableau de syndrome occlusif (douleur abdominale, arrêt des matières et des gaz, vomissements avec un ballonnement abdominal) évoluant depuis 36 heures. A l'examen, on retrouvait une bonne coloration des conjonctives et muqueuses, la température était à 37,6^°^C, la pression artérielle à 110/65 mm Hg et le pouls à 96 pulsations/mn. L'abdomen était distendu avec un tympanisme et une résistance élastique sans signes de péritonite. Les orifices herniaires étaient libres et le toucher rectal sans particularité. L'examen biologique notait un taux d'hémoglobine à 15,6g/dl, les globules blancs à 11200/µl, l'azotémie à 0,55g/l. La radiographie de l'abdomen sans préparation (ASP) montrait des images de niveaux hydro-aériques (Figure 1). Une laparotomie médiane sous ombilicale débordant en sus ombilicale a été réalisée. A l'exploration les anses intestinales étaient distendues en amont d'une portion iléale étranglée dans la fossette supravésicale. Cette zone incarcérée est située à 70 cm de la jonction iléo-cæcale. La réduction, faite après section du collet herniaire, découvrait une anse en souffrance mais viable (Figure 2 A). Une résection partielle du sac herniaire et fermeture simple par du vicryl 2/0 en points séparés ont été effectuées (Figure 2 B). Les suites opératoires étaient simples avec sortie à J4.

## Discussion

Les hernies supravésicales prennent naissance aux dépend des fossettes supravésicales qui sont situées entre les reliquats de l'ouraque et les artères ombilicales [3, 8]. Elles se forment en dedans des artères ombilicales et s’épanouissent le plus souvent dans l'espace de Retzius où elles s'engagent latéralement dans la paroi abdominale antérieure formant ainsi les hernies supravésicales externes ou latérovésicales [4, 5, 8]. Plus rarement le sac se forme autour de l'espace qui entoure la vessie pour former la hernie interne supravésicale [8, 9]. Les hernies internes ne représentent que 0,5 à 1% des toutes les causes d'occlusion intestinale. La hernie interne supravésicale constitue une forme rare de hernies internes et son incidence demeure difficile à apprécier [1–7]. Elle atteint préférentiellement l'homme de plus de 50ans [4, 5]. Le diagnostic préopératoire fortuit, peut être exceptionnellement évoqué devant des signes scannographiques [2, 6, 9]. Dans notre observation, comme pour de nombreux auteurs [1, 2, 4, 5], le diagnostic de l'occlusion intestinale aigue était facile, cependant la hernie supravésicale était une découverte per-opératoire. Même si le diagnostic préopératoire reste inhabituel, certains auteurs avaient rapporté des cas déjà évoqués par la tomodensitométrie abdominale avant l'intervention chirurgicale [1, 6, 9]. La suture simple du sac herniaire est suffisante pour certains auteurs et permet de prévenir les récidives [1, 3]. Dans notre cas, nous avons réalisé une résection de l'excès du sac et une fermeture en points séparés. La hernie interne supravésicale est de bon pronostic [1] et il dépend surtout de la précocité du diagnostic et de la prise en charge de l'occlusion intestinale. Certains auteurs avaient réalisé une résection intestinale à cause de la nécrose [3, 9].

## Conclusion

La hernie supravésicale interne est rare. Son diagnostic est le plus souvent établi en peropératoire lors de la prise en charge d'une occlusion intestinale aiguë. Son traitement simple par la fermeture du sac permet d’éviter les récidives. Devant une occlusion intestinale aiguë, il faut avoir à l'esprit que la hernie interne supravésicale peut en être une cause inhabituelle.
